# How has big data contributed to obesity research? A review of the literature

**DOI:** 10.1038/s41366-018-0153-7

**Published:** 2018-07-18

**Authors:** Kate A. Timmins, Mark A. Green, Duncan Radley, Michelle A. Morris, Jamie Pearce

**Affiliations:** 1 School of Sport and Exercise Science, University of Lincoln, Lincoln, NE USA; 2 School of Environmental Sciences, University of Liverpool, Liverpool, UK; 3 School of Sport, Leeds Beckett University, Leeds, UK; 4 Leeds Institute for Data Analytics, School of Medicine, University of Leeds, Leeds, UK; 5 Centre for Research on Environment, Society and Health, School of Geosciences, University of Edinburgh, Edinburgh, UK

## Abstract

There has been growing interest in the potential of ‘big data’ to enhance our understanding in medicine and public health. Although there is no agreed definition of big data, accepted critical components include greater volume, complexity, coverage and speed of availability. Much of these data are ‘found’ (as opposed to ‘made’), in that they have been collected for non-research purposes, but could include valuable information for research. The aim of this paper is to review the contribution of ‘found’ data to obesity research to date, and describe the benefits and challenges encountered. A narrative review was conducted to identify and collate peer-reviewed research studies. Database searches conducted up to September 2017 found original studies using a variety of data types and sources. These included: retail sales, transport, geospatial, commercial weight management data, social media, and smartphones and wearable technologies. The narrative review highlights the variety of data uses in the literature: describing the built environment, exploring social networks, estimating nutrient purchases or assessing the impact of interventions. The examples demonstrate four significant ways in which ‘found’ data can complement conventional ‘made’ data: firstly, in moving beyond constraints in scope (coverage, size and temporality); secondly, in providing objective, quantitative measures; thirdly, in reaching hard-to-access population groups; and lastly in the potential for evaluating real-world interventions. Alongside these opportunities, ‘found’ data come with distinct challenges, such as: ethical and legal questions around access and ownership; commercial sensitivities; costs; lack of control over data acquisition; validity; representativeness; finding appropriate comparators; and complexities of data processing, management and linkage. Despite widespread recognition of the opportunities, the impact of ‘found’ data on academic obesity research has been limited. The merit of such data lies not in their novelty, but in the benefits they could add over and above, or in combination with, conventionally collected data.

## Introduction

There has been growing interest in the potential of ‘big data’ for enhancing our understanding of a wide array of societal challenges including in medicine and public health. Facilitated by advances in computing hardware, software and networking, big data have been heralded as a powerful new resource that can provide novel insights into human behaviour and social phenomena. Despite the broad excitement and interest, there is no single agreed definition of big data. However, it is widely accepted that the greater volume, complexity, coverage and speed of availability of the observations and variables are critical components [1, 2]. In contrast, conventional, or ‘small’, data (e.g. from trials, cohorts or surveys), tend to be produced in more constrained ways using sampling strategies that restrict the scope (e.g. number of questions), size (e.g. number of respondents) or temporality (e.g. number of time points).

Big data generation tends to strive to: be comprehensive, often capturing full populations; have high temporal and/or spatial resolution; be interlinked and connected across different data resources with common fields to enable unique identification; and be dynamic and adaptive to allow new and greater quantities of data to be readily appended [3]. Connelly et al. [2] make the useful distinction between data that are ‘made’ and that which are ‘found’. ‘Made’ data include information collected to investigate a defined hypotheses; whereas ‘found’ data have been collected for alternative (often non-research) purposes, but could include potentially valuable information for research. The sources and production of ‘found’ data include, but are not limited to, online activities (e.g. social media, web searches), commercial transactions (e.g. in-store purchase from supermarkets or bank transactions), remote physiological sensors (e.g. heart-rate monitors) or environmental sensors (e.g. GPS, satellite data).

With increasing volumes and greater access to data in electronic formats, it is unsurprising that researchers are beginning to apply big data to key concerns including mental health [4], infectious disease [5] and healthcare [6]. In the field of obesity research, there is a long history of using routine data sources to track the prevalence of the disease, as well as identify risk factors. Supplementing this with new forms of data has potential to broaden our understanding of obesity, bringing together information from different facets of environment and behaviours. Although obtaining, analysing and disseminating big data has potential to benefit society, there are also a number of possible risks [3, 7], including challenges relating to data governance and methodological robustness. There has not yet been an attempt to review the current applications of big data to obesity-related research.

The aim of this paper is to review the contribution of ‘found’ data (adopting Connelley et al’s distinction) to obesity research, and consider the implications for the future of big data in this field. We focus on data that have been repurposed for research, rather than data originally designed for research or health monitoring purposes (such as health register or birth cohorts), because these sources of data offer new opportunities and challenges compared to conventional ‘made’ research data. Our intention is to review the nature and scope of the research that is emerging, and describe the benefits and challenges encountered.

## Methods

The aim of this review was illustrative, rather than to provide an exhaustive examination of obesity research examples. We developed a narrative, rather than systematic, review that identifies and collates research in which ‘found’ data have been adopted to address obesity-related concerns. From a scoping of the literature in November 2016, informed by activities within the ESRC Strategic Network for Obesity meetings (reference pending), we identified six categories of data: retail sales, transport, geospatial, commercial weight management data, social media, and smartphones and wearable technologies. These data categories are described in the Results.

Database searches were conducted between January and April 2017 (MEDLINE, PsycINFO, SPORTDiscus) using search terms such as: obesity, diet*, physical activity, body mass index, big data, commercial data, loyalty card, smart ticket, smart metr*, point of sale, tax*, purchas*, social media, crowd sourc*, app, mobile phone, cell phone. We only considered articles published in English in peer-reviewed academic literature, which described original research, and that used data sets not originally intended for research purposes. Outcomes considered relevant included measures of obesity, as well as dietary or physical activity outcomes. Search updates were run in September 2017, and articles were also found through citations and expert recommendation.

For each data category, we collated details from relevant studies to describe the data used, how and why they had been used, and the benefits and limitations of using them. We then considered as a whole the extent to which these data had contributed to obesity research to date.

## Results

An overview of the examples found in the literature can be seen in Table 1, including a brief summary of the added value and limitations of each data type. These are described in more detail below.

**Table 1:** Summary of the implementation, value and challenges of ‘found’ data in obesity research

Data type	Examples of data	Research aims	Value added over conventional data	Challenges
Retail sales	•Retailer data sets [11–15] •Checkout scanners [8, 9] •Consumer marketing panels [10] •National-level industry data [16, 17]	•Monitoring nutrient availability •Examining patterns in sales and national BMI estimates •Evaluating policy/ intervention impact on food/nutrient purchases	•Wider population coverage •Reduction of burden and cost •Improved ecological validity •Automation of data collection •Extension and timeliness of data collection period—lends itself to quasi-experimental studies •Reduction of subjective self-reporting	•Sampling: - finding appropriate comparison/control data for longitudinal analyses - representativeness •Difficulties linking to nutrition data •Incomplete data •Access and cost
Transport	•Road/transport cameras/sensors [23] •Bicycle hire data [25–27] •Driver licensces [24]	•Investigating ecological association between motorised transport and obesity •Evaluating impact of active travel schemes (e.g. bike hire)	•Enhanced detail—times, spatial locations, frequencies, duration, cost, multiple modes •Objective (not self-report) •Low respondent burden •Long-term trends, time series analysis	•Lack of complementary data on other relevant behaviours and outcomes
Commercial weight management programmes	•NHS referred patients to commercial programmes [28] •Self-referred members of weight-loss groups [29, 31] •Direct-to-consumer internet programme [30] •Family-based programmes [32, 35]	•Real-world service evaluations •Investigating referral: pathways, uptake •Determining attrition rates •Examining variation in outcomes by participant, family, neighbourhood and programme characteristics	•Scale •Improved ecological validity •Longitudinal data over several years	•Data accessibility, quality, completeness and representativeness •Commercial sensitivities require consideration •Ethical issue around consent for sharing data with 3rd parties
Geospatial	•Web mapping platforms [37, 38] •Remote sensing [39, 40] •Consumer databases [36] •National mapping agencies [36] •Global positioning systems (GPS)	•Measuring features of the built environment •Assessing exposure to food outlets or leisure facilities •Correlating neighbourhood walkability with obesity	•Finer resolution data: - spatially - temporally •Increased scope—national/international level •Minimised costs Increased access	•Lack of complementary data on other relevant behaviours and outcomes
Social media	•Twitter [42–47] •Facebook [48–51] •Reddit [52] •Foresquare [53] •Instagram [53] •Strava [54] •Online forums [55]	•Geographically locating posts about certain behaviours •Network analysis of message spread •Likes and shares as proxies for behaviours •Characterising discussions around obesity/weight •Measuring use of fast food outlets •Estimating physical activity or BMI from forum self-reports	•Offers social perspective •Low cost •Insight into social networks, which are difficult to measure •International scope •Immediacy	•Sample bias and representativeness •Validity of data (presence of ‘bots’) •Discrepancy between online and offline personalities
Smartphones and wearable technologies	•Step count apps [66] •Fitness log apps [63–65] •Integrated GPS data [65, 67] •Gaming apps [68, 69] •Weight-loss app [62]	•Exploring activity patterns across populations •Evaluating infrastructure changes on active travel •Assessing impact of active gaming apps •Characterising successful users of weight-loss app	•Wide-scale coverage, international •Lower respondent burden •Passive and continuous data collection	•Sample bias and representativeness •Lack of control on data acquisition—technical issues, consistency of user behaviour •Potential variability in device accuracy •Ethical issue around sharing data •Processing challenges due to size of data sets

### Retail sales data

#### What are the data?

Perhaps the earliest usage of ‘found’ data for obesity research involves the examination of retail sales data. Product sales data have long been collected by retailers to monitor transactions. Data can be taken directly from barcode scanners [8, 9], consumer marketing panels [10], retailer data sets [11–15] or national-level industry data [16, 17]. More recently, these data have been linked to individual-level information (e.g. age, sex, address) using store loyalty cards [18].

#### What has the data been used for?

Published studies have had varied purposes: monitoring nutrient or food intakes at a population level [8, 16, 17], ascertaining national or regional nutrient availability [19], comparing ‘vice’ purchases online versus in store [15], or evaluating the impact of policies or interventions (e.g. changes to benefits (food stamps) [12], nutrition labelling [20], taxation [10, 14] or public health campaigns [13]). Some studies have looked at the association between sales and aggregate-level outcomes (e.g. national-level BMI estimates [16, 17]), or examined longitudinal patterns in sales [10, 13, 14].

#### What do they add over and above conventional data?

There appear to be three motivations for using this type of data: wide coverage (e.g. population level [16, 17]); high ecological validity [14, 15] and benefits of automation [8, 21]. Conventional dietary assessment is often criticised as: burdensome, reliant on self-reports, expensive and typically only practical for use during a short window of time. Automatically collected sales data could reduce both respondent [22] and researcher [21] burden, and potentially minimise self-report errors [9, 19, 21]. Automation should also be considerably more cost-effective [8, 9, 11, 21, 22], enabling the collection of longitudinal and more timely data.

Sales data may be particularly useful for quasi-experimental evaluations of policy, where conventional randomised controlled trials (RCTs) may not be possible, and timely, longitudinal data are crucial. For example: Nikolova et al. [20] investigated the effect of point-of-sale nutritional information on consumer behaviour; Andreyeva et al [12] assessed the impact on nutrient purchases following revisions to federal food provision in the US; Colchero et al. [10] monitored panel members’ drinks purchases before and after the introduction of a tax on sugar-sweetened beverages in Mexico; Schwartz et al. [13] examined supermarket sales of sugary drinks before and during a campaign to reduce consumption and compared sales to those outside the community; and Silver et al. [14] looked at the impact of a tax on sugar-sweetened beverage consumption before and after a tax was implemented in Berkeley, California.

#### What are the limitations?

All studies identified issues in coverage, as they were only able to access data from certain supermarket chains [13, 14] or panels, which were not representative [10]. In addition, purchases of food and drinks do not necessarily equate to dietary consumption [8, 12, 22]. Furthermore, no studies have yet been able to link to individual-level health outcomes. Several authors also described problems with the quality of the data, for example, missing data due to technical faults or inconsistencies in recording [9, 14, 19, 21]. This is compounded by the dynamic nature of the retail food market [21, 22]. Data linkage was one of the main challenges identified in this type of study.

Quasi-experimental studies, whilst high in ecological validity, are unable to isolate the causal mechanism given the many potential confounders, and researchers struggle to find appropriate comparison data; some studies compared to counterfactual data (i.e. consumption predicted on the basis of pre-tax trends), which come with a number of assumptions [10, 14] and do not generate results demonstrating causal relationships.

A final challenge identified is the relationship with commercial partners. There is a concern that these data sets may prove cost-prohibitive for research purposes [22], and that their use may be restricted by non-disclosure agreements [22] or confidentiality worries [19]. Difficulties initiating partnerships or with finding partners with appropriate data collection were also described [14].

### Transport

#### What are the data?

Transport monitoring has long involved the collection of data on mode and volume of transport to aid in planning and infrastructure. Collection of transport data is increasingly sophisticated and new technologies can offer novel insights into travel and lifestyle behaviour as well. For example, on-board sensors within vehicles to monitor vehicle performance can provide data on travel patterns. External sensors along transport networks such as roads or public transport are also increasingly more common both for monitoring transport flows and in the fields of urban informatics. The popularity of smart card systems for public transport systems also presents an opportunity for obtaining information on destinations, routes and transport modes, and may include additional information about individuals such as socio-demographic characteristics.

#### What have the data been used for?

There were few applications utilising such data within obesity-related research. Some studies have used aggregated data sources to explore patterns associated with obesity. For example, Lopez-Zetina et al. [23] used data collected from the ‘Highway Performance Monitoring System’ on traffic flow data for public roadways in the US to investigate the ecological association between areas with greater motorised transport usage (vehicle miles of travel) and obesity prevalence. US driver licence data have also been proposed as a potentially useful opportunity as they contain information on height and weight [24]. Other applications have compared the impacts from the introduction of city-based bicycle hire schemes, by analysing usage data from cycle hire stations [25]. Some studies have also used these data as inputs to simulation models to estimate the impacts on health outcomes [26, 27].

#### What do they add over and above conventional data?

Transport data often include explicit information about spatial location. We know little about the activity spaces and environments that individuals engage within their daily lives and these data can illuminate the role of urban structure, utilisation of services, or engagement with green space. Conventional research exploring their associations with obesity tend to rely on simple approximations of these concepts, whereas new forms of data can provide a more valid and objective picture of exposure. They additionally present greater detail on how individuals are engaging with different modes of transport. The rise of private motorised transport has been touted as one important driver of obesity trends [23]. These data can therefore help to improve our understanding of physical activity from transport options that conventional data are unable to cover.

#### What are the limitations?

A key criticism is that many data sources only contain journey information, with little additional information about lifestyle behaviours or socio-demographic characteristics. Similar to retail sales data (above), the link between what is measured and the relevant behaviour can only be assumed or extrapolated. For example, knowing that an individual travelled from point A to point B can only inform us about the direction of their travel, and not the impact of travel on physical activity or dietary behaviours, nor the wider impact of an intervention. Data linkage is therefore important to be able to unpick these complex interactions to provide robust explanations for obesity-related behaviour.

### Commercial weight management data

#### What are the data?

This category refers to data that are provided by commercial weight management programmes. Weight management programmes routinely collect data not for research but as a standard part of their service provision. The intended use of the data may vary, possibilities including: client orientated feedback (e.g. self-monitoring), continuous service improvement (e.g. to monitor adaptations to programme content/delivery) and, if the service is being delivered as a procured provision, to monitor contractual targets (e.g. reporting key performance indicators). Data sets are often substantial in terms of participant numbers, and include information on individual characteristics (e.g. socio-demographic factors), engagement with the programme (e.g. enrolment, attrition or service usage) and weight outcomes.

#### What have the data been used for?

Commercial data provide the opportunity for independent real-world service evaluations. For instance: Ahern et al. [28] reported outcomes for 29,326 participants attending Weight Watchers NHS Referral Scheme between April 2007 and October 2009; Finley et al. [29] examined 60,164 men and women, aged 18–79 years, who enrolled in the Jenny Craig Platinum programme between May 2001 and May 2002; Johnson et al. [30] investigated Nutracheck, a direct-to-consumer Internet weight-loss programme; Stubbs et al. [31] reported the short-term outcomes of 1,356,105 self-referred, fee-paying adult participants of Slimming World groups joining between January 2010 and April 2012; and Fagg et al. [32] assessed outcomes associated with participation in a family-based weight management programme (MEND 7–13, Mind, Exercise, Nutrition..Do it!) for childhood overweight in 21,132 referred or self-referred children.

#### What do they add over and above conventional data?

These outcome evaluations provide important insight given that many large-scale programmes being used to treat obesity have not had their effectiveness formally evaluated using recognised research methodologies (e.g. RCTs). Further, even when programmes have been rigorously evaluated under trial conditions, programme effectiveness observed within controlled settings may differ to outcomes in real-world contexts [33, 34].

The data also provide the opportunity to consider a variety of research questions that are commonly not addressed within conventional effectiveness trial research designs or are beyond the scope of such evaluations. For instance, the data collected are often substantial in terms of numbers of participants: Fagg et al. [32, 35] were able to investigate: who is referred to, who started and who completed a child weight management intervention when delivered at scale; whether the socio-demographic characteristics of children attending the intervention matched those of the eligible population; changes in BMI observed under service conditions with those observed under research conditions; and how outcomes of the intervention varied by participant, family, neighbourhood and programme characteristics—all of which was enabled by the large-scale implementation of the intervention.

The wide-reaching scope of data in terms of participants also could allow investigation into hard-to-reach populations who are typically under-represented in conventional research. For example, Fagg et al. were able to explore patterns in programme usage by ethnicity and socioeconomic status—both of which are important to increase our understanding of health inequalities. Combining with other data sources, such as social media, transport and geospatial data, could present further useful insights, for example, by exploring relationships between the environment and programme outcomes.

#### What are the limitations?

Similar to the literature on retail sales data (see above), it is recognised that data accessibility, quality, completeness and representativeness must be addressed. Commercial sensitivities also need to be considered, as do ethical issues surrounding consent for data use and achieving appropriate levels of information security, confidentiality, and privacy, particularly given that individual-level data may be involved.

### Geospatial

#### What are the data?

Geospatial refers to data in which the location of objects across environments are stored with a spatially explicit dimension. They include the location of services (e.g. healthcare facilities, restaurants), the layout of road networks, or features of the built environment (e.g. parks, woodland). Data may be accessed through retail databases, national mapping agencies, satellite technology or web mapping platforms (e.g. Google Maps, OpenStreetMap).

#### What have the data been used for?

Geospatial data have been used to measure different features of the built and natural environment. Many studies have calculated simple counts of retail locations such as fast food outlets as a measure of exposure. For example, consumer and national agency data sources were used to create open access measures of accessibility to retail opportunities including fast food outlets or leisure services [36]. Other mapping services such as ‘Google Street View’ [37, 38] and remote sensing [39, 40] have also been used to develop virtual audits of environmental features which are then correlated to measures of obesity.

#### What do they add over and above conventional data?

Where locational information has been collated using conventional approaches (e.g. field audits, surveys), they are often restricted in multiple ways. Data may be collected separately by locale, resulting in gaps in spatial coverage, discrepancies in the information provided by locale, or a lack of joined-up inclusion of data limiting the ability to undertake national-level analyses. They may appear temporally infrequent, and while annual data may be appropriate, services such as Google Maps can allow finer temporal resolution for nuanced analyses. Conventional data sources may also impose costs or licensing arrangements of use of data or in accessing data.

#### What are the limitations?

The main drawback is similar to that identified for transport data (above). Typically, geospatial data are fairly basic containing only the location and type of object. To build up a comprehensive view of how humans interact with these objects, we need to know much more. For example, while identifying the location of fast food outlets is valuable, also important are details on types of food sold, opening hours, business turnover, and the nature of in-store marketing and product placementLinkage of data to other sources may increase their usefulness in obesity research—for example, tracking individuals’ movements within and interactions with the environment using GPS-enabled smartphones (see below).

### Social media

#### What are the data?

Social media are computer-assisted technologies that facilitate the creation of virtual networks connecting individuals and allowing the sharing of information. Their use has grown since the beginning of the twenty-first century and are embedded in the everyday lives of many people with, for example, 63% of UK adults using online social networks daily [41]. The ways in which individuals interact with these services are stored by their providers and can be made available to researchers.

#### What have the data been used for?

Twitter data represented the majority of studies utilising social media sources. Twitter is an online platform where users can write and share short posts of (at the time of writing) 140 characters or fewer (and may include geographical location when sent using mobile devices). Unlike other social media platforms, Twitter makes a portion (~1%) of its data freely available. Studies typically focused on using descriptive statistics to examine patterns of what was posted. Some studies used geotagged tweets to produce geographical measures of behaviours including dietary behaviours [42–44], physical activity [44, 45] or happiness/wellbeing [42, 46]. These were then correlated with data on obesity rates or the density of fast food outlets. Other examples include using social network analysis to explore how messages about childhood obesity spread between individuals [47].

Other social media platforms have been less commonly utilised. Facebook data on posts shared and interests followed (identified using ‘likes’) were used as proxies for behaviours and opinions/perceptions surrounding obesity [48–50]. One study examined correlations between these data and ecological measures of obesity [51]. Other examples included using Reddit posts to characterise discussions about weight loss [52], utilisation of fast food outlets using Foresquare and Instagram [53], Strava data to explore physical activity behaviours [54] or self-reporting of body weight on an online forum [55].

#### What do they add over and above conventional data?

With individuals opting to increasingly document their lives through digital platforms, social media data offer the potential to form intricate understandings of opinions, interactions with objects, locations and other individuals [56]. There is a paucity of data on social networks of individuals, and collecting ‘made’ data on the topic is both intensive and costly. Social media data offer cheaper and more comprehensive data on the issue, which can facilitate more in-depth studies on human interactions (particularly international interactions which are rarely considered). This is important given that it has been previously demonstrated that social networks have important roles in understanding obesity [57].

#### What are the limitations?

Few studies have engaged with the representativeness of social media data. For example, studies using Twitter data are purely describing patterns within Twitter users only, who disproportionately represent younger age groups [58], or even within just those Twitter users who allow geotagging (estimated at just over 1% [59]). Moving beyond single platforms will not only improve the generalisability of findings, but also open up opportunities for understanding how individuals engage with the increasing digitalisation of life. Linked to this notion of representativeness, we cannot ignore the increasing proportion of ‘bots’ among social media sites. Bots are automated social media accounts which post content with the aim of mimicking the behaviours of individuals. As such, they may contribute data to research, introducing bias to analyses [60]. Furthermore, our online personalities may not approximate who we are ‘offline’ [61].

### Smartphones and wearable technologies

#### What are the data?

Smartphones are increasingly pervasive—estimates suggest almost 70% of US adults owned a smartphone in 2015 [62]. With ever more sophisticated technology, many smartphones now incorporate a range of sensors and logs that open up opportunities for continuous collection of data in free-living environments. Often used alongside smartphones, linked devices, such as wrist-worn activity monitors or heart-rate monitors (wearable technologies), are used to track a user’s behaviour and are often used to supplement ‘life-logs’. Data may be made available from device or app manufacturers.

#### What have the data been used for?

Studies have typically used smartphone data to describe physical activity outcomes, such as step counts, GPS movements or logged journeys. In this way, activity patterns have been explored across populations, temporally or spatially [63–65]. There is some overlap here with geospatial data, where smartphone-integrated GPS can be triangulated with app data to describe the use of neighbourhoods or environments. As many smartphones and apps are widely utilised, the data can be used to make international comparisons, for example, correlating activity levels (using step counts) with national obesity trends [66]. Smartphone data have also been used to evaluate interventions: Heesch et al. [67] examine cycling behaviour before and after infrastructure changes. Other uses include assessing the influence of smartphone games on physical activity (Pokémon GO [68, 69]), or characterising successful users of a weight-loss app (Lose It! [62]).

#### What do they add over and above conventional data?

A key advantage of smartphone data is the wide-scale coverage, often international. This enables research that is broad in geographic scope, and large data sets offer additional analytical possibilities by being split into ‘training’ and ‘validation’ subsets [62]. In addition, where data recording is ‘passive’ and continuous, there is a lower respondent burden than many conventional methods, with potential benefits for participant adherence and longitudinal data collection. Apps which require users to actively log information (i.e. the data are non-passively generated) often include prompts and reminders, and thus may offer similar advantages as recognised for Ecological Momentary Assessment [70]. Incorporating GPS also allows the collection of geographically specific information. Several authors identified that sampling or inferential issues could be at least partially overcome by triangulating smartphone data with conventional research data to offer reassurances in terms of representativeness and validity.

#### What are the limitations?

A key issue is sampling: only those individuals who own a particular app, device or model of smartphone will be included in the data. Furthermore, authors cited concerns about the lack of control on data generation, as participants may not consistently carry their phone with them and switched on [64, 66]. Missing data due to technical reasons were also common, for example when signal or battery cut out [64, 71]. Smartphones are also unable to capture activities where people are unlikely to have their phone on them, such as contact sports or swimming. Finally, user behaviour may be both measured by and influenced by the smartphone app or wearable device itself, with potential repercussions for the interpretation of findings.

## Discussion

This paper provides an overview of how ‘found’ data have been used in obesity research to date. The narrative review highlights the variety of uses in the literature, with contrasting types of data and varied research questions: from describing the built environment, to exploring social networks, estimating nutrient purchases or assessing the impact of interventions. Importantly, each of the described studies has attempted in some way to use this data to infer behaviours associated with energy balance (diet and physical activity) or to understand the context in which obesity-related behavioural decisions are made. In the ensuing discussion, we offer a summary of the opportunities highlighted by the literature. The intention is to illustrate areas of interest and promise, rather than attempt a full critical evaluation of the use of data in these studies.

### Opportunities for big data research

The examples identified in this review demonstrate four significant ways in which ‘found’ data can complement the more conventional ‘made’ data: firstly, in moving beyond constraints in scope (in terms of coverage, size, and temporality); secondly, in providing objective, quantitative measures where conventional research has had to rely on self-reported data; thirdly, in reaching populations that have proven difficult to access with conventional research methods; and lastly in its potential for evaluating real-world interventions. We discuss each of these opportunities in turn.

Firstly, many of the examples of ‘found’ data described here are remarkable in their broad scope and coverage. The constraints of conventional ‘made’ data have provided much of the impetus for exploring the potential of repurposed data. Advocates of ‘found’ data suggest that automation could reduce the burden of data collection [8, 21]. It follows that a reduction in burden would allow more data to be collected over a longer period, both because of reduced costs and also due to reduced participant burden. This was particularly evident in the retail sales literature. RCTs or evaluations could automatically be updated with long-term data without having to collect a lot of information from participants.

Secondly, automated data collection could make an important contribution where conventional methods rely on self-reported information. There is much research that has documented the systematic biases, which have plagued obesity-related research through individuals misreporting their weight, dietary intake, or physical activity [72]. Other important factors that have proven traditionally difficult to measure include environmental characteristics which are theorised to have a role in the aetiology of obesity [73, 74]. Data from transport and geospatial sources, in particular, could offer a means of capturing environmental features, although work may still be needed to develop meaningful, validated metrics. Given the suspected multi-faceted influences on obesity [75], the ability to measure specific aspects of the aetiology of obesity will help to build a more complete picture of its determinants. Thus, the opportunities afforded through objective data automatically collected from ‘found’ data could revolutionise our understanding of many complex areas [56]. The ability to quantify increasingly complex scenarios could also prove invaluable for predictive explorations, such as investigating system dynamics or agent-based modelling [76].

Thirdly, we can leverage the broad scope of these big data to explore hard-to-reach populations that conventional data are unable to access or provide precise estimates on [56, 77]. For example, the Health Survey for England 2014 [78], one of the largest and most comprehensive sources of data on health-related behaviours (*n* = 10, 041), included only 1332 non-White individuals. Understanding the role of ethnicity, a key non-modifiable factor in obesity research, becomes problematic here. Big data can help, and can be extended to smaller groups as well. Linked to this, the growing interest in understanding the heterogeneity of obesity [79] can be improved through capturing more nuanced data to examine the interactions between risk factors and behavioural characteristics.

Finally, ‘found’ data provide a key opportunity for quasi-experimental research, by which we mean natural experiments that assess the impact of a policy or intervention. Examples from our review included evaluations of commercial weight management programmes [28–31, 35], and assessing the impacts of events as diverse as infrastructure changes (e.g. new cycle routes) [67], popular gaming apps [68, 69], changes to taxation on obesity-related commodities (e.g. sugar-sweetened beverages) [10, 14] or local campaigns [13, 20]. These examples illustrate the value of repurposed data for assessing real-world change. For example, without ‘found’ data, conventional methods would have required a cohort recruited well before an intervention or policy was implemented, with longitudinal collection of data. Using repurposed data that have been collected consistently for an adequate period of time, on the other hand, means that timely, longitudinal patterns can be explored, without a costly and lengthy lead-in. Although necessarily observational, and whilst there may be difficulties in finding appropriate comparators, the implications for the evaluation of public health (and other) policies are obvious. A number of these quasi-experimental studies adopted a combined approach [14, 67], complementing the use of ‘found’ data with a more conventional research design, which illustrates perhaps one of the ways the limitations of big data could be addressed.

Quasi-experimental studies were rare for some types of data—namely travel, geospatial and social media data—and published studies in these categories predominantly focussed on descriptive, rather than causal, questions. This could be a promising area for future research: if causal investigation could broaden across multiple levels of determinants, such as those described by the Social-Ecological Model [80], from the individual to the structural, the ability to look at multiple factors across multiple scales might better allow us to begin to unpack the complexity of obesity development and prevention. Mapping the possible data sources that would allow this is an important first step to realising multi-level research, and forms the basis of the subsequent paper from our network (reference pending).

These opportunities are not without challenges. Many of the limitations described in this review are not necessarily new. For example, ‘found’ data sets typically comprise convenience samples [56]. However, the use of ‘found’ data also throws up some distinct challenges, such as:

- ethical and legal questions around access and ownership of data
- commercial sensitivities and potential costs
- lack of control over data acquisition
- questions over attributional adequacy—big data are often mono-thematic with great depth but limited breadth—and the clinical relevance of measurements
- finding appropriate comparators
- new skills and capabilities necessary for data processing, management and linkage.

These challenges have been well described by colleagues in relation to other health outcomes [2, 7, 56], and a further detailed exposition of these limitations is not possible here. However, addressing these issues will be of vital importance to enable utilisation of these data as well as considering the profound implications in terms of validity.

Accessibility to each data type was a common barrier to the usage of big data in obesity-related research. Many data types were held by industrial partners who are not always willing to permit researchers to use this information (although there are numerous examples where commercial data are being utilised for research purposes) or the costs associated with usage were prohibitive. Recently, multiple trusted third parties have been established to provide indirect access to such data and help bridge such gaps between industry and researchers (e.g. Consumer Data Research Centre in UK). Social media and geospatial data were more often openly available, hence the preponderance of studies utilising this type of information. Time and cost were minimal issues in reducing access, and when compared to traditional data, found data can be more efficient in terms of time and cost for data collection [3]. While there is no natural order to the quality or reliability of found data, we advocate that the pitfalls of ‘big data research’ are no different from traditional research. Any data should be assessed for its representativeness or bias no matter how big or small. For example, while Twitter data were the most common data source encountered in the review, the key limitation of this information is that it is not generalisable to whole population [56].

It is perhaps as important to comment on the gaps in data usage. The literature described here demonstrate initial forays into big data usage in the field of obesity. However, there are examples of ‘found’ data usage in other research areas that were notably absent in the obesity literature. For example, we did not observe any studies, which made use of ‘found’ data in the form of physiological or biological measurements, although measurement is becoming possible through smartphone technologies (e.g. peripheral capillary oxygen saturation or heart rate) [81]. This highlights that there are many future opportunities in exploring untapped data sources.

### Limitations of the review

This review was not intended as an exhaustive examination of obesity research using ‘found’ data; rather, the aim was to illustrate the opportunities afforded by such data. This was important to demonstrate how and why such forms of data have been used in obesity research to date, and provide some key opportunities as to what can be achieved with such data in the future. It is also important to note that the scope of this synthesis was limited to academic literature.

The focus here was on ‘found’ data, repurposed for research, rather than on ‘big data’. Big data are not synonymous with ‘found’ data. However, much of the data described as ‘big’ has been repurposed from non-research-specific sources. This, we believe, is where much of the opportunity of big data lies: where data are collected anyway, its scope in terms of coverage, timeliness and automation could make a real, fresh contribution to the ways we are able to measure behavioural and environmental variables. By focussing on ‘found’ data, we hoped to identify its potential as well as the concomitant challenges, regardless of size, ‘big’ or ‘small’. Some of the studies described would not be considered ‘big’ by most, yet these smaller examples help to reveal or address potential problems with validity or data processing. In many cases, it is apparent that these need to be resolved at this smaller scale before upscaling to larger data sets.

Our focus has meant that some undeniably ‘big’ data sets are absent from our narrative: health registers and genetic databases were beyond our scope, yet their potential in obesity research is apparent. Many of the advantages described for ‘found’ data also apply to these data types: for example, health registers offer great scope in terms of volume and longitudinal and geographical coverage. However, ‘found’ data are an as yet under-utilised source of information, and many of the opportunities have yet to be exploited. ‘Found’ data also come with unique challenges to processing, storage and interpretation, given that they are created outside a research environment, and are therefore worthy of separate attention.

## Conclusions

This paper has shown the limited extent to which ‘found’ data have been employed in academic obesity research to date, as well as describing the unique contribution such data can add to conventional research. The examples from the literature demonstrate how the merit of such data lies not in their novelty, but in the benefits they add over and above, or in combination with, conventionally collected data. However, alongside these new opportunities, there are new and distinct challenges. There is still a need to investigate ways to combine these new forms of data with conventional research to increase confidence in their validity and interpretation.

Despite widespread recognition of the opportunities across a broad spectrum of disciplines and data types, the potential of ‘found’ data has not yet been fully realised, and the impact on academic obesity research has been limited. In part, this may be due to limited data access, or even a lack of awareness about the data that may be available. The aim of the next paper from the ESRC Strategic Network for Obesity (reference pending) is to highlight the potential sources of data for further research of this type, many of which are as yet untapped.
